# Influence of the Molar Mass and Concentration of the Polyvinylpyrrolidone on the Physical–Mechanical Properties of Polylactic Acid for Food Packaging

**DOI:** 10.3390/polym17162218

**Published:** 2025-08-14

**Authors:** Ivan Restrepo, Eliezer Velásquez, María Galotto, Abel Guarda

**Affiliations:** 1 Faculty of Engineering, University Alberto Hurtado, Santiago 8340576, Chile; 2 Packaging Innovation Center (LABEN), University of Santiago of Chile (USACH), Obispo Umaña 050, Santiago 9170201, Chile; 3 Faculty of Engineering, University of Santiago of Chile (USACH), Santiago 9170201, Chile

**Keywords:** polylactic acid, polyvinylpyrrolidone, packaging

## Abstract

Improving the end-of-life performance of polylactic acid (PLA) for food packaging requires strategies that enhance biodegradability, solubility, and dispersibility without compromising essential material properties. PLA-based films were produced by melt extrusion using polyvinylpyrrolidone (PVP) as a hydrophilic modifier, aiming to enhance the water uptake and affinity of PLA, which may potentially lead to faster environmental degradation. Two PVPs with distinct molar masses at varying concentrations were used to investigate their effects on the structural, thermal, mechanical, optical, and barrier behavior of the films. Thermal analysis revealed a slight depression in glass transition temperature, more evident in blends with low-molecular-weight PVP10, indicating increased chain mobility and partial miscibility. A two-step degradation process with extended thermal decomposition profiles was observed upon the inclusion of PVP. SEM and ATR-FTIR analyses confirmed enhanced dispersion and non-covalent interactions in PVP10-based blends, in contrast to the pronounced phase separation and micro-voids observed in PVP40-based systems. Mechanically, films containing 5 and 10 wt.% of PVP10 retained tensile strength and stiffness, whereas PVP40 led to embrittlement. Optical properties were modified by increasing the PVP content, resulting in greater opacity and color differences, which potentially offer benefits for light-sensitive packaging. Altogether, PLA films containing 5 and 10 wt.% of PVP10 demonstrated the most favorable balance between water affinity-oriented design and packaging-relevant performance.

## 1. Introduction

The transition toward sustainable packaging has intensified the search for biodegradable polymers that can replace conventional plastics without compromising functionality. Among the available options, polylactic acid (PLA), an aliphatic polyester derived from renewable feedstocks such as corn starch and sugarcane, stands out for its biodegradability, optical clarity, and compatibility with standard thermoplastic processing [1,2]. Despite these advantages, PLA’s application in food packaging, its low solubility and dispersibility, and low biodegradation are limited at the end of service life [3,4].

To enhance the performance and environmental responsiveness of PLA, blending with functional hydrophilic polymers has emerged as a promising approach. One such additive is polyvinylpyrrolidone (PVP), a biocompatible, water-soluble polymer widely used in pharmaceutical and biomedical fields. PVP offers several desirable features, including high moisture uptake capacity, strong hydrogen bonding potential, and the ability to disrupt hydrophobic matrix packing [5,6]. When incorporated into PLA, PVP can increase the system’s overall hydrophilicity, potentially accelerating biodegradation by facilitating water diffusion and microbial access [7]. However, the introduction of hydrophilic domains may also impact visual appearance, mechanical strength, thermal properties, and barrier properties, critical attributes for packaging functionality.

Previous investigations into PLA–PVP blends have shown encouraging results regarding drug release modulation, wettability, and processability, especially when prepared by electrospinning or solution casting [8,9]. Nevertheless, most of these studies use a single grade of PVP, leaving unexplored the impact of its molar mass on the structure–property relationships of melt-extruded PLA-based films, which is a closer approach to the industrial process. It has been reported that the molar mass of the polymers complementing the PLA blend plays a crucial role in determining the polymer’s behavior, phase morphology, mechanical performance, crystallinity, and thermal properties, as observed in blends of PLA and polyvinyl alcohol [10].

This work aims to fill this knowledge gap by systematically evaluating PLA–PVP films produced via melt cast extrusion using two types of PVP with distinct molar masses at varying concentrations (5, 10, and 15 wt.%). Through a comprehensive analysis of their structural, morphological, thermal, mechanical, and optical properties, as well as water vapor permeability, this study aims to assess how PVP’s molar mass modulates the properties of PLA-based packaging materials. Ultimately, this research contributes to the development of potential biodegradable film systems with enhanced end-of-life behavior by tuning water affinity, while maintaining the physical properties required for safe and effective food packaging.

## 2. Materials and Methods

### 2.1. Materials

Polylactic acid (PLA, Ingeo™ 2003D) was supplied by NatureWorks LLC (Plymouth, MN, USA) in pellet form, with a D-isomer content of 1.4% and a melt flow index of 6 g 10 min^−^^1^ (210 °C/2.16 kg). Polyvinylpyrrolidone of different molecular weights, PVP10 ($\bar{M_{w}}$ = 10,000 g mol^−^^1^) and PVP40 ($\bar{M_{w}}$ = 40,000 g mol^−^^1^), were purchased from Sigma-Aldrich (Louis, MO, USA). All materials were vacuum-dried at 50 °C for 24 h before use.

### 2.2. Preparation of PLA–PVP Films

Polylactic acid–polyvinylpyrrolidone (PLA–PVP) films were produced at concentrations of 5%, 10%, and 15% of PVP of different molar masses selected for their commercial availability, PVP10 and PVP40, by extrusion in a Labtech Scientific LTE-20–40 twin-screw extruder (Samutprakarn, Thailand). Prior to extrusion, the PLA and PVP were dried at 50 °C for 24 h to remove water that could cause hydrolytic degradation of PLA during extrusion. Then PLA and PVP were blended until homogenization before being added to the extrusion hopper. The temperature profile used was 190 °C to 210 °C from feed to die at 45 rpm with a torque of 50%. A flat film die was attached to the extruder head, enabling the direct production of continuous films. A neat PLA film was also processed under the same conditions as the control. The resulting films had a thickness of 100 ± 10 µm. Samples were coded as PLA-XPVPY, where X is the weight percentage of PVP in the blend PLA-PVP, and Y is 10 or 40 for PVP with a molar mass of 10,000 g mol^−^^1^ or 40,000 g mol^−^^1^, respectively.

### 2.3. Characterization of the Films

#### 2.3.1. Scanning Electron Microscopy (SEM)

The surface morphology of cryo-fractured films was examined using a TESCAN Vega 3 SEM (Czech Republic) at 10 kV. Samples were gold–palladium-coated using an Anatech Hummer 6.2 sputter coater (Hayward, CA, USA) with a field of vision < 500 μm.

#### 2.3.2. Attenuated Total Reflectance–Fourier Transformed Infrared Spectroscopy (ATR-FTIR)

Structural analysis of the samples was performed to determine thermal stability using a Bruker Alpha IFS 66V (Ettlingen, Germany) attenuated total reflection (ATR) Fourier transform infrared spectroscopy (FTIR) coupled to a Bruker Platinum attenuated total reflection (ATR) diamond crystal. Twenty-four scans were performed over a wavelength range of 4000 cm^−^^1^ to 360 cm^−^^1^ with a resolution of 4 cm^−^^1^. Each spectrum was analyzed using Opus v.7.0 software.

#### 2.3.3. Differential Scanning Calorimetry (DSC)

The thermal properties of the materials were obtained using a Mettler DSC-822e differential scanning calorimeter (Schwarzenbach, Switzerland). Briefly, 6 mg of sample was placed in aluminum capsules and subjected to three thermal scans: (i) 30 °C to 220 °C (first heating), (ii) 220 °C to 30 °C (cooling), (iii) 30 °C to 220 °C (second heating), with a heating/cooling rate of 10 °C min^−^^1^ under a nitrogen atmosphere (50 mL min^−1^). From this analysis, the glass transition (T_g_), melting (T_m_), and cold crystallization (T_cc_) temperatures, as well as the cold crystallization (ΔH_cc_) and melting (ΔH_m_) enthalpies, were determined during the second heating. The crystallinity of the materials was calculated using Equation (1):

(1) $$X_{c}=\frac{{∆H}_{m}}{{∆H}_{100}\cdot X_{PLA}}$$

where X_c_: crystallinity (%); ∆H_m_: melting enthalpy (J g^−1^); ∆H_100_: melting enthalpy of 100% crystalline PLA (93 J g^−1^) [11]; X_PLA_: mass fraction of PLA in the blend.

#### 2.3.4. Thermogravimetric Analysis (TGA)

The thermal stability of the materials was investigated using a Mettler TGA/DSC 1 (Schwarzenbach, Switzerland) thermogravimetric analysis system. For this purpose, 8 mg of sample was placed in alumina capsules and heated from 30 °C to 800 °C at a rate of 10 °C min^−^^1^, under a nitrogen atmosphere and with a flow rate of 50 mL min^−^^1^. From this analysis, the start of degradation (T_onset_) and maximum degradation rate (T_d_) temperatures were determined using the Mettler Toledo STARe software.

#### 2.3.5. Color and Opacity Index

The color difference (ΔE) was obtained as the average of four measurements along the film, using UltraScan VIS HunterLab equipment under D65 illumination and a 10° observer angle, and the CIELab scale. Coordinates L*, lightness, a*, which measures from red to green tones, and b*, which measures the changes between yellow and blue, were determined. ΔE is calculated through Equation (2) as the color variation in the PLA–PVP blend film with respect to the 100% PLA control film. Additionally, the opacity was determined using the same equipment on a black and white background, in accordance with ASTM D1746.

(2) $$∆E=\sqrt{\left[{\left(∆L\right)}^{2}+{\left(∆a\right)}^{2}+{\left(∆b\right)}^{2}\right]}$$

#### 2.3.6. Tensile Test

The effect of PVP incorporation on Young’s modulus (E_t_), tensile strength (σ_M_), and elongation at break (ε_B_) of PLA was evaluated in a Zwick/Roell Proline BDO-FB 0.5 TH universal machine (Ulm, Germany) following ASTM D882. For this, ten specimens measuring 21 cm × 2.5 cm were prepared per sample and kept at a relative humidity of 50% RH and a temperature of 23 °C for 48 h. A 500 N cell, a jaw separation of 125 mm, and a separation rate of 12.5 mm min^−^^1^ were used. Tensile parameters were reported as an average of ten measurements.

#### 2.3.7. Water Vapor Permeability (WVP)

The water vapor transmission rate (WVTR) was determined according to ASTM F-1249 at 37.8 °C and 90% RH in a Mocon Permatran W 3/34 apparatus (Neuwied, Germany). The water vapor permeability coefficient (WVP) was calculated using Equation (3), where the WVTR was multiplied by the film thickness and then divided by the water vapor pressure difference between the two sides of the film, calculated as 0.9 × Pwater _vapor_ at 37.8 °C. The analysis was performed in duplicate.

(3) $$WVP=\frac{\left(WVTR)x(thickness\right)}{∆P}$$

#### 2.3.8. Statistical Analysis

Data analysis was carried out by variance analysis (ANOVA) and a Fisher’s LSD post-ANOVA test with a random experimental design and a significance level of *p* < 0.05, using Statgraphics Centurion 19 software. Significant statistical differences for a parameter among samples are indicated by different superscript letters.

## 3. Results and Discussion

### 3.1. Scanning Electron Microscopy (SEM)

**Figure 1:**
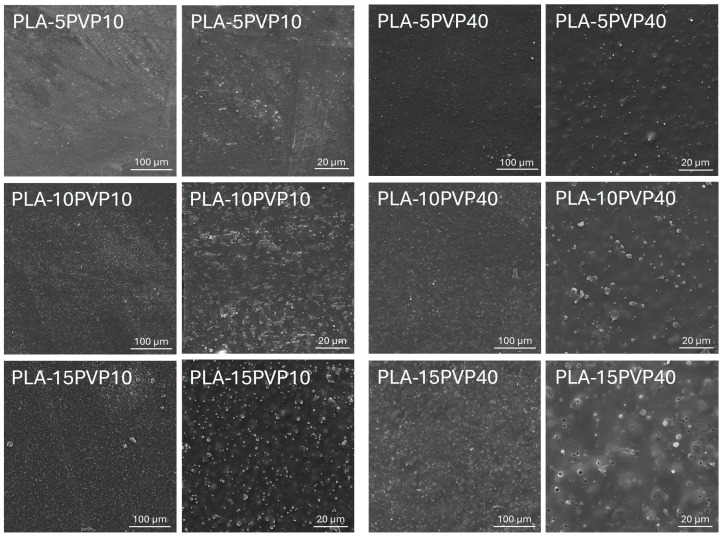
SEM micrographs of PLA films with 5, 10, and 15 wt.% PVP10 and PVP40. Magnifications 500X (first and third column) and 2000X (second and fourth column).

Figure 1 presents micrographs of PLA–PVP films obtained by SEM. They demonstrated the presence of PVP particles dispersed in the PLA matrix, the number and size of which increase with increasing PVP concentration. At 5% by weight of PVP40, a more homogeneous distribution of particles on the surface was evident, although the surface was rougher, due to the higher molar mass of PVP40 forming aggregates.

Furthermore, it was observed that increasing the PVP concentration resulted in greater homogeneity in the particle density on the surface of the films, attributed to improved dispersion and mixing of the polymers during film formation. However, surface void formation was detected at high PVP40 concentrations, associated with a lower miscibility of high-molar-mass PVP particles with PLA at this concentration. This led to low interaction at the PLA–PVP interface, triggering the formation of cracks, separation zones, and detachment of the PVP particles. These morphological features were further confirmed by the comparative analysis of PLA–PVP films containing PVP10, which displayed smoother and more continuous surfaces across all concentrations, particularly at 5–10 wt.%, indicating good interfacial compatibility and effective dispersion within the PLA matrix. Conversely, films with PVP40 exhibited increasingly irregular and porous structures, consistent with phase separation phenomena and insufficient chain-level miscibility, especially at 15 wt.%. This compromised structural integrity is consistent with observed reductions in mechanical properties and increases in optical opacity, which will be explained in the following sections. Surface roughening and defects have been reported in PLA–PVP systems depending on theophylline concentration as an additive in filaments formed for drug release materials [6].

### 3.2. Attenuated Total Reflectance–Fourier Transform Infrared Spectroscopy (ATR-FTIR)

**Figure 2:**
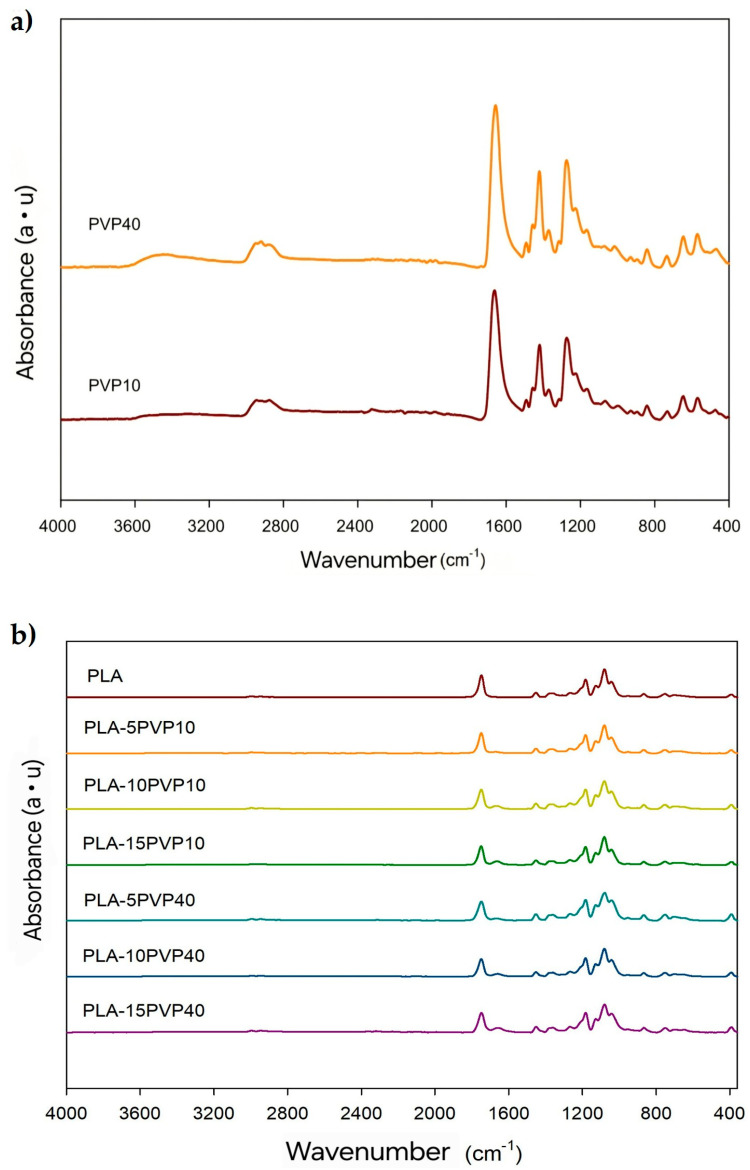
ATR-FTIR spectra of (**a**) PVP10 and PVP40 polymers and (**b**) PLA and PLA–PVP films.

Figure 2a shows the characteristic peaks of PVP for both PVP10 and PVP40. The band associated with the stretching of the C-N bond at 1274 cm^−1^ and the vibration of the CH_2_ group at 1420 cm^−1^ were detected. The absorption peak was attributed to the vibration of the C=O bond at 1663 cm^−1^, while the vibration of the asymmetric stretching of the C-H bond was detected at 2945 cm^−1^. The absorption band around 3400 cm^−1^ is associated with the vibration of the O-H group, resulting from the absorption of water by PVP, which is a hydrophilic polymer. Despite being oven-dried, this material exhibits high moisture absorption [12,13,14,15,16].

Figure 2b shows the characteristic peaks of PLA, such as the small bands at 2996 cm^−^^1^ and 2946 cm^−^^1^ corresponding to the stretching of the C-H bond of the methyl group (CH_3_) and the vibration of the carbonyl group (C=O) around 1750 cm^−^^1^. Likewise, bands visualized at 1452 cm^−^^1^, 1382 cm^−^^1^, and 1359 cm^−^^1^ were associated with the bending of CH_3_ and the symmetric and asymmetric deformation vibrations of the C-H bond present in the methyl groups of PLA, respectively [17]. On the other hand, the presence of PVP10 or PVP40 in the PLA–PVP films was verified by the presence of a band at 1664 cm^−^^1^ attributed to the vibration of the C=O bond of the pyrrolidone group of PVP, which became more intense with the PVP concentration. Additionally, a broadening of this band was observed in the wavenumber range of the carbonyl groups with increasing PVP concentration, which may indicate a greater interaction between the carbonyl groups of the pyrrolidone units with the terminal hydroxyl groups of the PLA chains through hydrogen bond interactions. The other characteristic bands of PVP could overlap with the bands of PLA [5]. No significant shifts were observed in the position of the PLA carbonyl band, which reinforces the absence of chemical reactions such as transesterification or covalent bond formation during melt blending. This observation supports previous findings that PLA–PVP blends are physically mixed systems stabilized by non-covalent interactions, such as hydrogen bonding [5,7]. Such structural stability is beneficial in food packaging applications, as it helps preserve the inherent biodegradability and safety profile of the materials.

### 3.3. Differential Scanning Calorimetry (DSC)

**Figure 3:**
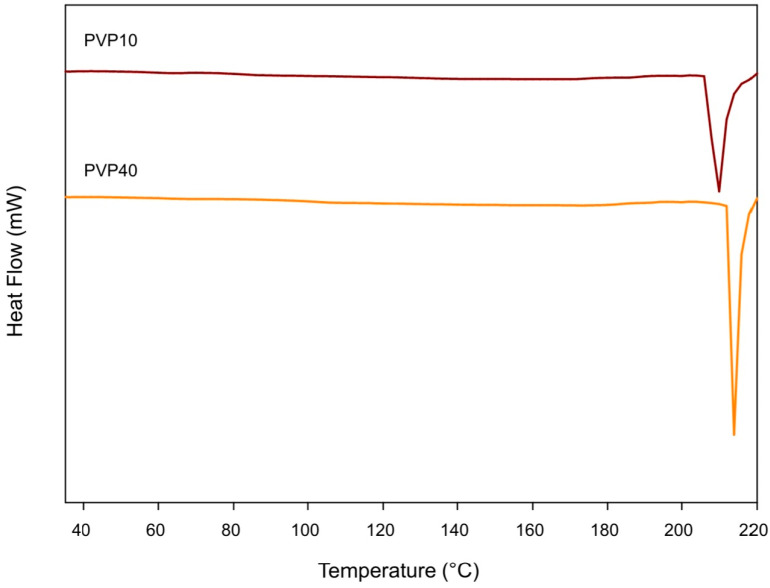
DSC first heating thermograms of the PVP10 and PVP40.

**Figure 4:**
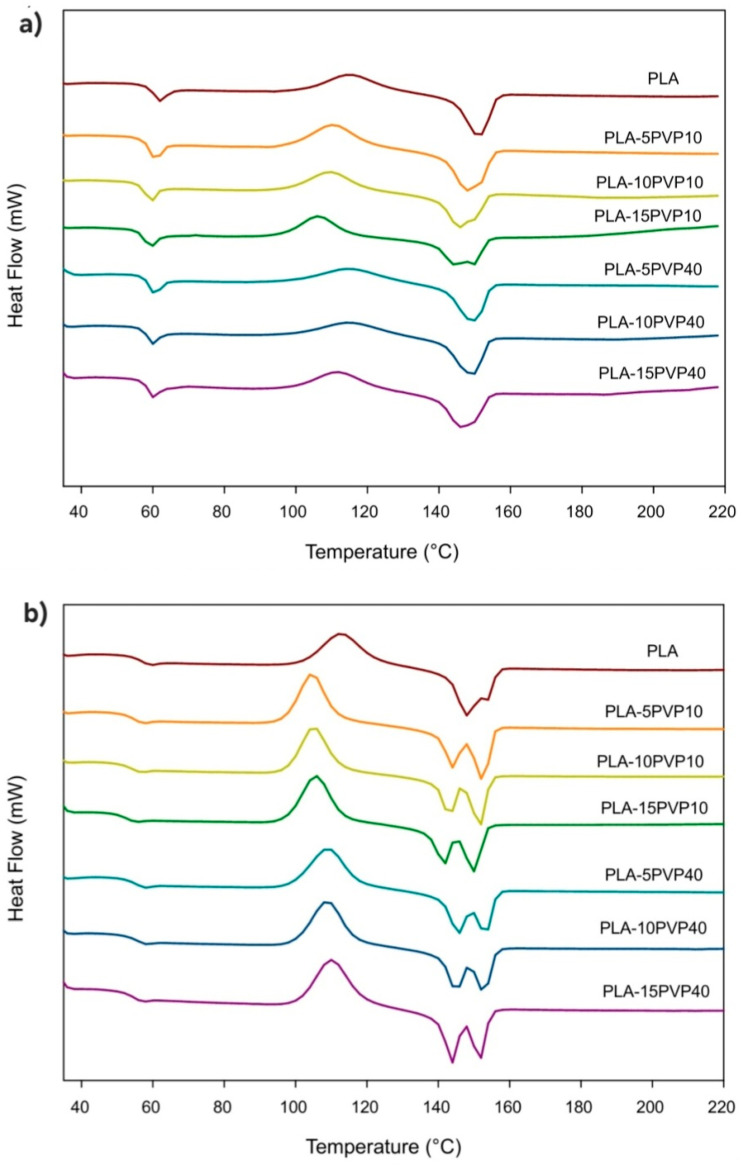
DSC thermograms of the PLA–PVP films: (**a**) first heating and (**b**) second heating.

**Figure 5:**
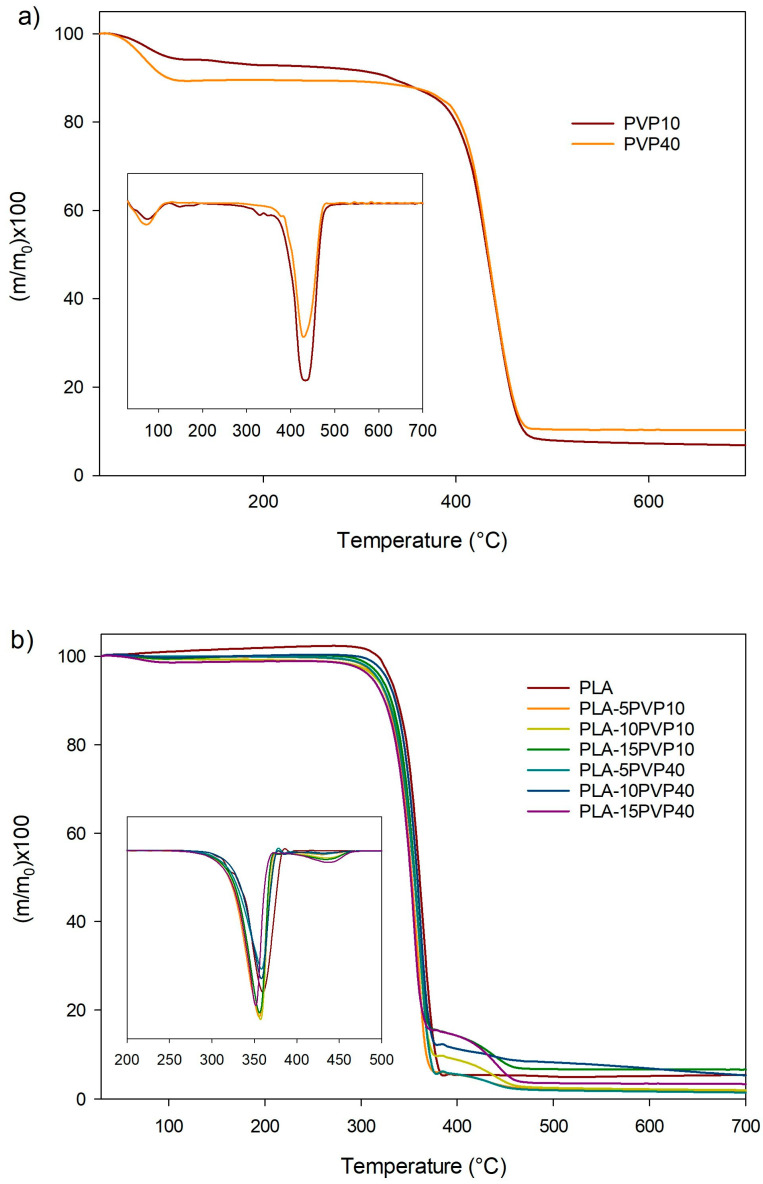
TG and DTG curves of (**a**) PVP and (**b**) films of PLA and PLA–PVP blends. Inserts corresponds to DTG curves being Y axis: d(m/m_o_)/dT.

Figure 3 presents the DSC thermograms of the first heating of PVP. Figure 4a,b shows the DSC thermograms of the PLA–PVP and PLA control films from the first and second heating, respectively, considering that no crystallization transition was observed during cooling to DSC conditions. Table 1 and Table 2 show the corresponding DSC parameters. The absence of a clear glass transition in PVP can be attributed to (i) its high degree of hydration, resulting from its high affinity for moisture, which reduces its T_g_ to very low temperatures or may even go undetected, or (ii) it has a wide glass transition range, typically between 50 °C and 175 °C, depending on the molar mass, moisture content, and presence of plasticizers [18,19]. Thus, the precise detection of glass transitions in PVP is complex because it could also be closer to, or overlap with, the mass loss range or some decomposition, depending on the molar mass of PVP, which could be initiated after 210 °C but occurs significantly after 300 °C (Figure 3 and Figure 5a).

PLA exhibits a glass transition at 60 °C, cold crystallization at 115 °C, and melting at 52 °C, in accordance with reports in the literature (Table 1 and Figure 4) [20]. However, when PVP is incorporated, a plasticizing effect is observed, evidenced by a slight decrease in T_g_, T_cc_, and T_m_. This effect becomes statistically significant at high PVP concentrations for PVP10 due to its lower molar mass, which accentuates its plasticizing effect (Table 1). Furthermore, the incorporation of PVP induced the formation of crystals with varying degrees of order, as evidenced by the presence of two well-defined melting peaks for PVP10 and at high PVP40 concentrations. This was clearly observed in all cases during the second heating, where the thermal history of the polymer blend was erased (Figure 4b). PVP produced the formation of two types of crystals with different lamellar thicknesses, with thinner crystals melting at a lower temperature, while thicker crystals melted at a higher temperature. The effect of a lower molar mass of PVP was revealed by a shift in T_cc_ to lower values in PLA–PVP10 blends at all concentrations.

Regarding the crystallinity of the films, the PLA–PVP films tended to increase their value relative to the PLA film crystallinity, although there were no statistically significant differences (Table 1). The crystallinity was highest for the PLA–PVP40 film, with a value of 10%, showing no clear trend with PVP concentration or molar mass.

In the second heating, after erasing the plastic’s thermal history, the same trends observed in the first heating were evident, but more pronounced, with a slight reduction in Tg and well-defined cold and melting crystallization peaks for all formulations. There was a tendency for crystallinity to increase with PVP up to 10% by weight; however, a reduction in the degree of ordering occurred due to controlled cooling with 15% PVP40. This indicates that ordering by controlled DSC cooling, in the absence of extrusion-induced chain direction, did not promote crystallization (Table 2).

The more defined dual-peak melting behavior was attributed to melt-recrystallization phenomena and heterogeneous nucleation induced by secondary components, as previously noted by [21]. Furthermore, the more evident reduction in T_cc_ in PVP10-based blends across all concentrations confirms the enhanced chain mobility introduced by lower-molecular-weight PVP, acting as a plasticizer. Notably, the modest increase in crystallinity during the first heating, followed by its decrease during the second heating, highlights the role of processing conditions and thermal history in defining final morphology, as reported by Restrepo et al. (2018) and Fazita et al. (2015) [10,20]. These results support the interpretation that while PVP promotes some level of crystallinity, the extruded film’s structure is primarily governed by melt flow orientation rather than crystallization kinetics alone.

### 3.4. Thermogravimetric Analysis (TGA)

Figure 5 presents the TGA curves of the samples, while Table 3 presents the TGA parameters of raw PVP and PLA–PVP films. PLA showed an onset of decomposition at 325 °C and showed a single peak for the maximum degradation rate at 362 °C (Table 3), which is consistent with the thermal parameters reported in the literature [22]. By incorporating PVP10 and PVP40, a reduction in thermal stability is observed, as evidenced by a decrease in T_onset_ and T_d_. Two peaks of maximum degradation were observed, the first between 350 °C and 357 °C associated with the decomposition of PLA, and the second between 433 °C and 441 °C attributed to the degradation of PVP (Figure 5b and Table 1). PVP10, as observed in Figure 5a and Table 3, presented four stages of mass loss occurring at 81 °C, 161 °C, 337 °C, and 438 °C. The first and second stages of mass loss are attributed to water evaporation due to the moisture content present in PVP10, given its hydrophilic character, and to the degradation of low-molecular-weight oligomers in PVP10. The third and fourth stages of mass loss are due to the structural decomposition of PVP10 chains [23,24]. In contrast, PVP40 exhibited a moisture-associated mass loss at 78 °C and a single degradation peak at 437 °C, suggesting a narrower molar mass distribution for PVP40.

On the other hand, a marked tendency toward a reduction in T_onset_ and T_d1_ was observed with increasing PVP40 concentration, although the PVP maximum degradation temperature in the blend increased (Table 3). However, it is important to note that the degradation temperature in PLA–PVP blends was significantly higher than the extrusion temperature required for the preparation of packaging materials. While the incorporation of PVP generally led to a reduction in T_onset_ possibly due to water absorbed by PVP, which could be favoring hydrolytic degradation of PLA, the presence of a distinct T_d2_ peak, especially in PVP40-containing formulations, indicates that thermal degradation extends into higher temperature domains, which may be attributed to the thermal stability of PVP40’s cyclic structure and its possible interaction with PLA degradation intermediates, similarly to the reported by Restrepo et al. for PLA–PVA blends [10]. These observations are consistent with prior reports on hydrophilic polymer additives that influence the decomposition profile through both physical and compositional effects.

### 3.5. Color and Opacity Index

**Figure 6:**
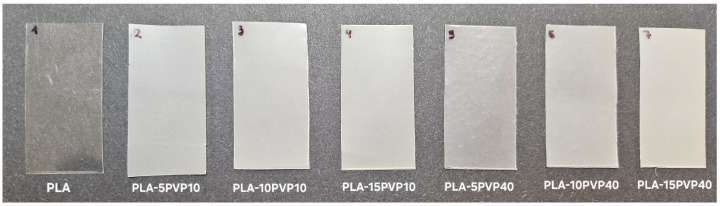
Photograph of the films showing opacity and color variation.

The color variation (ΔE) in the films was determined relative to the pure PLA film. The opacity and color coordinates, such as lightness (L*), which represents the lightness of the samples from 0 (black) to 100 (white), and a* and b*, where −a* is in the green direction, +a* is in the red direction, −b* is in the blue direction, and +b* is oriented toward the yellow color, are reported in Table 4. A photograph is shown in Figure 6.

Increasing the PVP concentration increased color variation, as PVP produces a whitish color with a slight yellowish tint. This is also supported by the a* and b* parameters, where increasing PVP concentrations led to more greenish (lower a* values) and yellow (higher b* values) hues. PVP10 caused greater color variation; however, the color change values were similar at 10% and 15% by weight, regardless of the molar mass of the PVP.

Regarding opacity, it was observed that opacity increased with the addition and increasing concentration of PVP in the PLA–PVP blend films, which tended toward greater crystallinity compared to the PLA film. However, there was no statistically clear trend with PVP molar mass and composition in the concentration range studied.

### 3.6. Tensile Test

**Figure 7:**
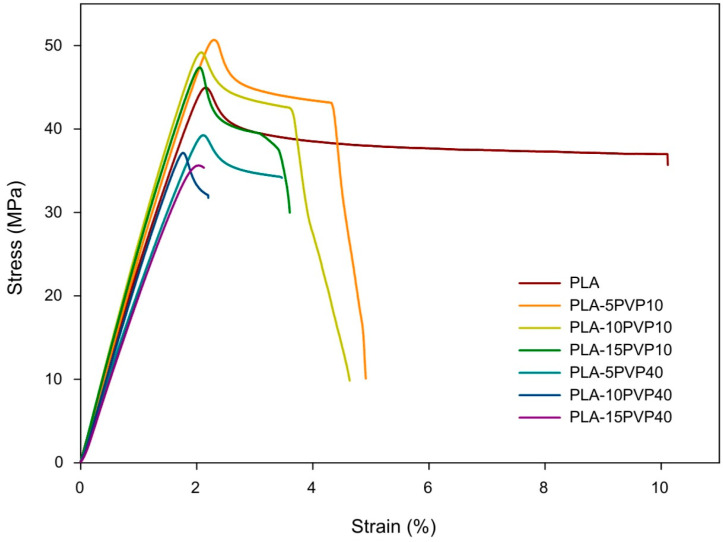
Stress vs. strain curves of the PLA and PLA–PVP films.

Figure 7 presents the stress vs. strain curves while Table 5 reports Young’s modulus (Et), ultimate tensile strength (σM), and elongation at break (εB) obtained for the PLA–PVP films. PLA exhibited the highest stiffness of the materials, supported by a higher Young’s modulus. Incorporating PVP10 at 5% and 10% by weight, no statistical differences were observed in the elastic modulus or tensile strength compared to pure PLA. However, adding 15% PVP10 significantly decreased stiffness and tensile strength. As for PVP40, its addition to the PLA matrix drastically and significantly decreased its mechanical properties, with no clear trend observed with respect to PVP concentration. This could be attributed to a more irregular surface of the films and the presence of holes, especially at 15% by weight, as shown in the SEM micrographs (Figure 1).

Regarding the percentage of strain at break, it was found that the incorporation of PVP resulted in a decrease in elongation, i.e., a reduction in the material’s ductility. This decrease in the percentage of deformation at break had a greater impact on the films with PVP40, which elongated 80% less than PLA at 15% by weight of PVP40, likely due to the greater presence of PVP agglomerates and holes in the film, which triggered the earlier breakage of the polymeric material.

The mechanical response of the PLA–PVP blends reflects not only intrinsic differences in polymer molar mass but also the impact of morphological heterogeneity and interfacial interactions. As revealed in SEM analysis, the rougher, more porous surfaces of PVP40-based films likely contribute to premature crack initiation and act as stress concentrators, which explains their markedly reduced elongation and strength. In contrast, the relatively smooth and homogeneous morphology of PVP10 blends at lower concentrations supports better stress distribution and cohesive failure modes, which may justify the retention of mechanical integrity in PLA–PVP10 (5–10 wt.%) systems. This interplay between morphological and mechanical properties reinforces the notion that PVP10, despite its slight plasticizing effect, preserves the mechanical framework of PLA when used at moderate concentrations.

### 3.7. Water Vapor Permeability (WVP)

**Figure 8:**
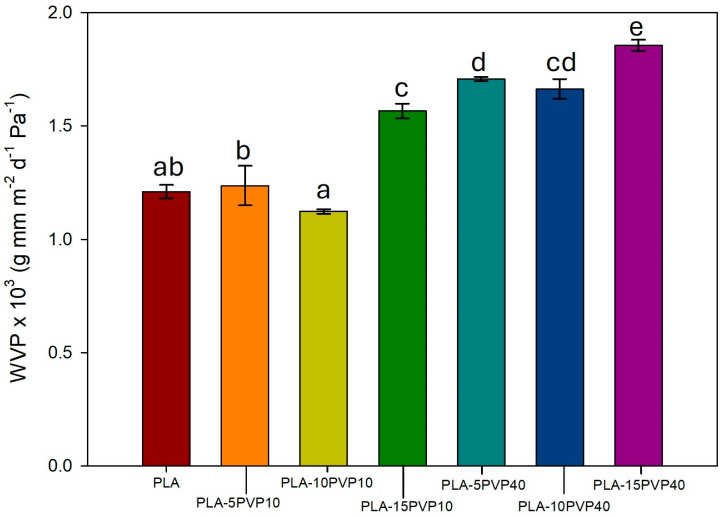
Water vapor permeability coefficient of PLA and PLA–PVP films. Significant statistical differences for a parameter among samples are indicated by different superscript letters (a, b, c, d, e), as determined by the ANOVA analysis with a significance level of *p* < 0.05.

Figure 8 shows that the incorporation of PVP into PLA films increased water vapor permeability due to its greater hydrophilicity compared to PLA. This increase was statistically significant at 15% PVP10 and all PVP40 concentrations, reaching maximum increases of 30% and 54% for PVP10 and PVP40-15 wt.%, respectively. The generation of voids in the PLA-15PVP40 films facilitated capillary water vapor permeation. Thus, the greater permeability of PLA at high concentrations of PVP could be attributed to a mechanism governed by capillary flow, rather than just diffusion through the polymer. Therefore, in blends with PVP10, the greater WVP can be attributed more certainly to the water affinity of the PLA–PVP-blend-based material.

## 4. Conclusions

The incorporation of PVP enhanced the hydrophilicity of the PLA matrix, which is desirable for promoting biodegradation and dispersion of PLA, resulting in a greater increase in water vapor permeability for PLA containing 15% PVP40 by weight. Furthermore, another property clearly modified was opacity and color variation, which increased with increasing PVP concentration. This could be potentially helpful for protecting light-sensitive products. Thermal analysis revealed that the onset of degradation decreased between 7 °C and 27 °C in the PVP concentration range studied, without a clear trend with respect to either concentration or molar mass of PVP; however, it extended the degradation range.

According to morphological analysis, PVP10 produced a more homogeneous surface than PVP40, which tended to form aggregates and generate porosity and voids in the film at 10 and 15 wt.%, thereby affecting its mechanical performance. Thus, for food packaging applications, PVP10 and low concentrations are more suitable, as homogeneous and smoother surfaces are obtained, advantageous for minimizing adverse effects on mechanical robustness, visual quality, and barrier performance.

Overall, PLA–PVP films with 15% PVP10 or 5% PVP40 achieved the most favorable balance between enhanced hydrophilicity, smoother and flawless surfaces, and retention of thermal stability and optical functionality, with minimal modification of the mechanical properties, resulting in lower Young’s modulus and, therefore, less rigid films than pure PLA. These findings underscore the importance of tailoring the additive molar mass of PVP to design biodegradable PLA–PVP films for potential use in sustainable food packaging systems. Considering that increasing the concentration of PVP results in higher water vapor permeability values at the expense of color variation, these more opaque, translucent materials have potential applications in achieving an organic, handcrafted look for biodegradable food packaging, a market with a growing niche of consumers.

## Figures and Tables

**Table 1:** Thermal properties from the DSC first heating.

Sample	T_g_ (°C)	T_cc_ (°C)	ΔH_cc_ (J g^−1^)	T_m1_ (°C)	T_m2_ (°C)	ΔH_m_ (J g^−1^)	X_c_ (%)
PLA	60 ± 0.1 ^c^	115 ± 0.2 ^d^	20.1 ± 0.6 ^c^	-	152 ± 1.1 ^c^	26.4 ± 0.3 ^b^	6.1 ± 0.9 ^abc^
PLA-5PVP10	59 ± 0.1 ^b^	110 ± 0.3 ^b^	19.1 ± 0.1 ^bc^	148 ± 0.2 ^b^	152 ± 0.1 ^c^	27.0 ± 1.3 ^b^	7.8 ± 1.6 ^cd^
PLA-10PVP10	58 ± 0.7 ^ab^	110 ± 0.4 ^b^	19.4± 1.1 ^bc^	147 ± 0.9 ^b^	151 ± 0.1 ^bc^	24.9 ± 2.1 ^ab^	5.8 ± 1.1 ^ab^
PLA-15PVP10	58 ± 0.1 ^a^	106 ± 0.1 ^a^	18.5 ± 0.2 ^b^	144 ± 0.1 ^a^	150 ± 0.2 ^ab^	25.2 ± 0.4 ^ab^	9.0 ± 0.8 ^bc^
PLA-5PVP40	59 ± 0.2 ^b^	115 ± 0.1 ^d^	18.3 ± 0.1 ^b^	-	149 ± 0.2 ^a^	23.3 ± 0.3 ^a^	6.0 ± 0.4 ^a^
PLA-10PVP40	59 ± 0.2 ^b^	115 ± 0.2 ^d^	17.1 ± 0.1 ^a^	-	149 ± 0.9 ^a^	23.8 ± 0.2 ^a^	8.3 ± 0.4 ^bc^
PLA-15PVP40	59 ± 0.4 ^b^	112 ± 0.1 ^c^	16.4 ± 0.2 ^a^	147 ± 0.7 ^b^	150 ± 0.4 ^b^	24.8 ± 0.1 ^ab^	10.6 ± 0.1 ^d^

Significant statistical differences for a parameter among samples are indicated by different superscript letters (a, b, c, d), as determined by the ANOVA analysis with a significance level of *p* < 0.05. (-) means that there was no record in the analysis.

**Table 2:** Thermal properties from the DSC second heating.

Sample	T_g_ (°C)	T_cc_ (°C)	ΔH_cc_ (J g^−1^)	T_m1_ (°C)	T_m2_ (°C)	ΔH_m_ (J g^−1^)	X_c,PLA_ (%)
PLA	57 ± 0.4 ^e^	112 ± 1.2 ^c^	25.8 ± 0.3 ^a^	148 ± 0.2 ^e^	154 ± 0.1 ^d^	30.7 ± 0.6 ^a^	5.9 ± 0.9 ^b^
PLA-5PVP10	55 ± 0.5 ^cd^	105 ± 0.7 ^a^	27.3 ± 0.2 ^ab^	144 ± 0.4 ^c^	152 ± 0.3 ^c^	34.4 ± 0.1 ^b^	8.1 ± 0.2 ^e^
PLA-10PVP10	54 ± 0.1 ^b^	105 ± 0.5 ^a^	28.4 ± 0.8 ^b^	143 ± 0.1 ^b^	152 ± 0.1 ^b^	34.5 ± 0.7 ^b^	7.5 ± 0.2 ^de^
PLA-15PVP10	52 ± 0.5 ^a^	105 ± 0.8 ^a^	29.3 ± 1.9 ^b^	141 ± 1.0 ^a^	149 ± 0.8 ^a^	34.1 ± 1.9 ^b^	6.1 ± 0.1 ^bc^
PLA-5PVP40	55 ± 0.1 ^cd^	109 ± 0.1 ^b^	27.5 ± 0.5 ^ab^	145 ± 0.1 ^d^	153 ± 0.1 ^cd^	33.3 ± 0.2 ^b^	6.8 ± 0.4 ^cd^
PLA-10PVP40	56 ± 0.2 ^d^	109 ± 0.1 ^b^	27.8 ± 0.1 ^b^	145 ± 0.2 ^cd^	152 ± 0.2 ^d^	33.5 ± 0.1 ^b^	6.8 ± 0.1 ^cde^
PLA-15PVP40	55 ± 0.1 ^bc^	110 ± 0.5 ^b^	28.6 ± 0.3 ^b^	144 ± 0.1 ^bc^	152 ± 0.1 ^b^	31.4 ± 0.4 ^a^	4.1 ± 0.9 ^a^

Significant statistical differences for a parameter among samples are indicated by different superscript letters (a, b, c, d, e), as determined by the ANOVA analysis with a significance level of *p* < 0.05.

**Table 3:** TGA parameters of the samples.

Sample	T_onset_ (°C)	T_d1_ (°C)	T_d2_ (°C)	T_d3_ (°C)	T_d4_ (°C)
PVP10	75	81	161	337	438
PVP40	62	78	-	-	437
PLA	325	-	-	362	-
PLA-5PVP10	305	-	-	353	433
PLA-10PVP10	300	-	-	355	438
PLA-15PVP10	311	-	-	354	438
PLA-5PVP40	307	-	-	356	435
PLA-10PVP40	318	-	-	357	441
PLA-15PVP40	297	-	-	350	440

**Table 4:** Color variation and opacity of the films.

Sample	L*	a*	b*	ΔE	Opacity
PLA	98.97 ± 0.01 ^d^	−0.22 ± 0.01 ^f^	0.40 ± 0.02 ^a^	-	10.50 ± 0.01 ^a^
PLA-5PVP10	98.95 ± 0.01 ^d^	−2.35 ± 0.04 ^b^	6.12 ± 0.10 ^c^	6.11 ± 0.11 ^b^	16.23 ± 0.10 ^c^
PLA-10PVP10	98.86 ± 0.03 ^d^	−3.57 ± 0.03 ^a^	9.31 ± 0.10 ^f^	9.52 ± 0.11 ^d^	19.08 ± 0.22 ^e^
PLA-15PVP10	98.91 ± 0.06 ^d^	−3.48 ± 0.14 ^a^	8.78 ± 0.40 ^e^	8.99 ± 0.43 ^d^	18.20 ± 0.29 ^d^
PLA-5PVP40	98.72 ± 0.01 ^c^	−0.84 ± 0.02 ^e^	3.37 ± 0.10 ^b^	3.05 ± 0.10 ^a^	14.45 ± 0.21 ^b^
PLA-10PVP40	97.47 ± 0.06 ^a^	−1.52 ± 0.02 ^c^	9.11 ± 0.21 ^ef^	8.94 ± 0.21 ^d^	21.98 ± 0.38 ^f^
PLA-15PVP40	97.78 ± 0.18 ^b^	−1.33 ± 0.09 ^d^	7.57 ± 0.82 ^d^	7.36 ± 0.84 ^c^	19.48 ± 0.61 ^e^

Significant statistical differences for a parameter among samples are indicated by different superscript letters (a, b, c, d, e, f), as determined by the ANOVA analysis with a significance level of *p* < 0.05. (-) means that there was no record considering the PLA as the control.

**Table 5:** Tensile parameters of films PLA and PLA–PVP.

Sample	E_t_ (N mm^2^)	σ_M_ (N)	ε_B_ (%)
PLA	2728 ± 185 ^d^	53.5 ± 4.3 ^c^	10.3 ± 1.9 ^d^
PLA-5PVP10	2682 ± 233 ^cd^	52.5 ± 3.6 ^c^	5.8 ± 2.0 ^c^
PLA-10PVP10	2752 ± 163 ^d^	50.8 ± 2.6 ^c^	4.2 ± 1.1 ^b^
PLA-15PVP10	2485 ± 263 ^bc^	42.3 ± 4.2 ^ab^	3.8 ± 0.9 ^b^
PLA-5PVP40	2226 ± 112 ^a^	41.2 ± 1.5 ^ab^	3.7 ± 0.8 ^b^
PLA-10PVP40	2466 ± 255 ^b^	43.5 ± 9.4 ^b^	2.0 ± 0.3 ^a^
PLA-15PVP40	2253 ± 367 ^a^	38.3 ± 6.8 ^a^	2.0 ± 0.2 ^a^

Significant statistical differences for a parameter among samples are indicated by different superscript letters (a, b, c, d), as determined by the ANOVA analysis with a significance level of *p* < 0.05.

## Data Availability

The data are contained within the article.
